# Water-Resistant Photo-Crosslinked PEO/PEGDA Electrospun Nanofibers for Application in Catalysis

**DOI:** 10.3390/membranes13020212

**Published:** 2023-02-08

**Authors:** Emanuele Maccaferri, Andrea Canciani, Laura Mazzocchetti, Tiziana Benelli, Loris Giorgini, Stefania Albonetti

**Affiliations:** 1 Department of Industrial Chemistry “Toso Montanari”, University of Bologna, Viale Risorgimento 4, 40136 Bologna, Italy; 2 National Interuniversity Consortium of Materials Science and Technology (INSTM), 50121 Florence, Italy; 3 Interdepartmental Center for Industrial Research on Advanced Applications in Mechanical Engineering and Materials Technology, CIRI-MAM, University of Bologna, Viale Risorgimento 2, 40136 Bologna, Italy

**Keywords:** polyethylene oxide, poly(ethylene glycol) diacrylate, electrospinning, blend, nanofiber, photoinitiator, photo-curing, crosslinking, water-resistance, catalysis

## Abstract

Catalysts are used for producing the vast majority of chemical products. Usually, catalytic membranes are inorganic. However, when dealing with reactions conducted at low temperatures, such as in the production of fine chemicals, polymeric catalytic membranes are preferred due to a more competitive cost and easier tunability compared to inorganic ones. In the present work, nanofibrous mats made of poly(ethylene oxide), PEO, and poly(ethylene glycol) diacrylate, PEGDA, blends with the Au/Pd catalyst are proposed as catalytic membranes for water phase and low-temperature reactions. While PEO is a water-soluble polymer, its blending with PEGDA can be exploited to make the overall PEO/PEGDA blend nanofibers water-resistant upon photo-crosslinking. Thus, after the optimization of the blend solution (PEO molecular weight, PEO/PEGDA ratio, photoinitiator amount), electrospinning process, and UV irradiation time, the resulting nanofibrous mat is able to maintain the nanostructure in water. The addition of the Au_6_/Pd_1_ catalyst (supported on TiO_2_) in the PEO/PEGDA blend allows the production of a catalytic nanofibrous membrane. The reduction of 4-nitrophenol (4-NP) to 4-aminophenol (4-AP), taken as a water phase model reaction, demonstrates the potential usage of PEO-based membranes in catalysis.

## 1. Introduction

More than 90% of commercialized chemical products, spanning from petroleum to agriculture, polymers, electronics, and pharmaceutical industries, derive from catalytic processes. Indeed, catalysis enables cost reduction, time saving, and a decrease in waste generation, making it a pivotal factor in developing a more sustainable chemical industry, as reported by the twelve principles of green chemistry [1,2,3]. Other aspects related to the concept of green chemistry are the process intensification and the process integration, that is, the combination of different steps such as reaction and separation in a single unit. This could be achieved by developing catalytic membranes and membrane reactors [4]. However, the industrial application of catalytic membranes is currently uncommon, mainly due to the need for membrane optimization [5]. In high-temperature conditions, inorganic membranes are the most diffused; they can be applied in several gas phase reactions, e.g., dehydrogenation reactions, methane–steam reforming, water/gas shift reactions, selective oxidations, oxidative dehydrogenations of hydrocarbons, and methane oxidation to synthetic gas [6]. In contrast, in low-temperature applications, such as in the case of fine chemical production, polymeric catalytic membranes are preferred due to a more competitive cost and easier tunability compared to inorganic ones [7].

Catalytic membranes in membrane reactors can carry out three different functions: i) extractor (selective removal of one product from the reaction mixture), ii) distributor (controlled addition of a reactant to the reaction mixture), and iii) contactor (intensification of the contact between reactant and catalyst). When used as contactors, since the catalyst is placed inside the membrane pores, they allow intense contact between the reactants, which flow convectively through the pores, and the catalyst, avoiding typical diffusion issues that occur in classical fixed-bed reactors. This setup either aims to achieve high catalytic activity to maximize conversion or high selectivity by operating on the contact time [4].

The polymeric catalytic membranes’ efficiency as contactors depends mainly on the pore dimension. The higher the exposed surface area, the higher the catalytic membrane activity; hence, in this context, nano-porous materials, such as nanofibrous membranes, would satisfy this need, provided that reagents are able to diffuse inside the polymeric nanofiber matrix and reach the catalyst. Indeed, the natural porosity driven by the nanofibrous membrane, together with the intrinsic flexibility of the fibers themselves and their ability to move one with respect to another, allows it to overcome common diffusion problems connected with the porosity of classic bulk membranes, while only the potential diffusion problems inside the nanofibers can negatively affect the catalytic activity.

Electrospinning is an efficient and easily scalable process for producing nanofibrous polymeric membranes characterized by nano-porosity [8], besides high versatility. Indeed, with the appropriate rational design of the membrane, it is possible to introduce additional functionalities, i.e., hydrophilicity/hydrophobicity and piezoelectric and self-cleaning properties [9,10,11]. Nanofibers are already exploited in several fields, such as filtration [12,13], tissue engineering [14,15,16], sensing [17,18,19], and catalysis [20,21,22]. Moreover, the technique allows the production of nano-reinforced nanofibers (e.g., with graphene and related materials, GRMs [23,24]) and blend/mixed nanofibers [25,26] to impart/tailor specific properties. In addition, nanofibers can even be post-processed to achieve the desired characteristics [27,28].

In catalysis, the main application of electrospun membranes is relegated to the production of ceramic nanofibers. In this case, a starting solution composed of a polymer matrix, a ceramic precursor, and metallic salt is electrospun to achieve a nanofibrous organic–inorganic membrane, which is then calcined both to remove the organic part and degrade the metal salt to obtain metal nanoparticles [29,30,31,32]. However, the polymeric component should be preserved to keep catalytic membranes’ physical and mechanical properties. Some works [33,34] report on the production of polymeric catalytic membranes via surface decoration of electrospun nanofibers, but this procedure involves several steps that are not easily compatible with a scale-up of the process. From this point of view, a more suitable way is the production of catalytic membranes incorporating the catalyst directly into the polymeric solution, which then will be electrospun, as reported by Bonincontro et al. [21]. The produced catalytic membranes work in the oxidation of 5-hydroxymethylfurfural (HMF) to 2,5-furandicarboxylic acid (FDCA); however, to obtain the best performance, the diffusion issue within the nanofibers should be minimized to enhance the ability to act as a contactor polymeric membrane. They demonstrated that the glass transition temperature (*T_g_*) of the polymer used for hosting the catalyst plays an important role in the catalytic behavior. It was found that Nylon 66-based nanofibers (*T_g_* ≈ 50 °C) work better than polyacrylonitrile (PAN) ones (*T_g_* ≈ 90 °C). Indeed, above the *T_g_*, the macromolecular chains acquire higher mobility, thus favoring the diffusion of small molecules (both reagents and products) toward and from the catalyst. The same work showed that the higher the reaction temperature is away from the polymeric matrix *T_g_*, the higher the production yield. Additionally, a polymer’s high affinity with the reaction solvent favors a facilitated diffusion of reagents within the fibers toward the catalyst. For the presented reasons, a polymer matrix promotes the contact between reagents and the catalyst when possessing (i) a *T_g_* below the reactor operating temperature, and (ii) a high affinity with the solvent but without dissolving it.

Poly(ethylene oxide), PEO, is a water-soluble polymer with a low *T*_g_ of around −70 °C [35] and a melting temperature (*T*_m_) near 60–70 °C [36]. The limited resistance at relatively low temperatures and its large dissolution in plenty of common solvents [32,33] prevents PEO usage in several application fields [37], such as in the catalysis of liquid phase reactions. Several strategies can be adopted to overcome these potential points of weakness; among others, crosslinking is a viable solution [38,39,40]. For example, poly(ethylene glycol) diacrylate, PEGDA, and trimethylol propane triacrylate, TMPTA, in addition to crosslinkers, have been demonstrated to be effective [41,42].

While PEO use is almost relegated to biomedical and healthcare applications [43,44,45,46,47,48,49], its usage in other fields is uncommon, such as in composite laminates for hindering delamination [36] and as phase change materials (PCMs) [50]. Even PEO application in catalysis is limited to a few reported cases [51,52,53]; however, it has never been shaped into nanofibers for catalytic purposes.

**Figure 1:**
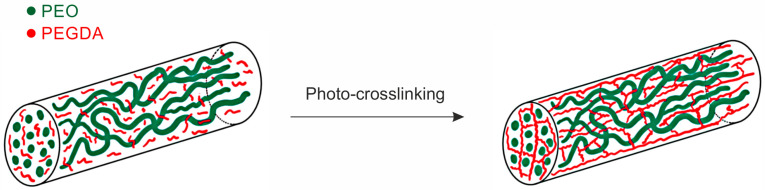
Schematic of PEO/PEGDA nanofiber, before and after PEGDA photo-crosslinking.

The present work aims to produce catalytic PEO/PEGDA nanofibrous membranes, via a single-needle electrospinning process, for their application in water phase and low-temperature reactions. The design concept is that PEO will allow the electrospinning of the PEO/PEGDA blend, guaranteeing, at the same time, membrane hydrophilicity, while PEGDA, after photo-crosslinking, will create a 3D scaffold around PEO chains making the overall membrane insoluble. Figure 1 illustrates the situation, on the macromolecular scale, of the PEO/PEGDA nanofiber before and after the covalent bonding between PEGDA oligomers thanks to photo-crosslinking.

Introducing a catalyst within the nanofiber should make it possible to produce a contactor-type polymeric catalytic membrane in which the two aforementioned characteristics (low *T*_g_ and high affinity with the solvent) are integrated. The catalytic membrane, containing Au_6_Pd_1_/TiO_2_ as the active phase, is used to reduce 4-nitrophenol (4-NP) to 4-aminophenol (4-AP), a water phase model reaction used here to demonstrate the potential use of PEO-based membranes in catalysis.

## 2. Materials and Methods

### 2.1. Materials

Poly(ethylene oxide) (PEO) with different molecular weights (M_w_ of 100, 400, and 1000 kDa) was used. Poly(ethylene glycol) diacrylate (PEGDA, M_n_ = 575 Da) was used as the oligomeric crosslinker. 2,2-Dimethoxy-1,2-diphenylethan-1-one (Ciba IRGACURE) was used as a photoinitiator (PI). All of the materials were purchased from Sigma-Aldrich (St. Louis, MO, USA) and used as received.

### 2.2. Preparation of Au_6_Pd_1_/TiO_2_ Catalyst, Plain PEO Solutions, PEO/PEGDA, and PEO/PEGDA/Catalyst Blends, Electrospinning, and Photo-Crosslinking

The Au_6_Pd_1_/TiO_2_ catalyst was prepared as reported by Lolli et al. [54] via the immobilization on the titania of the preformed bimetallic nanoparticles with Au:Pd ratio 6/1. Briefly, the Pd/Au colloid preparation procedure consisted of the dissolution of poly(vinylpyrrolidone), PVP, used as a nanoparticle stabilizer, and NaOH in water. The solution was then heated to 95 °C, the temperature at which β-d-glucose and an aqueous solution containing the metal precursors (HAuCl_4_ and PdCl_2_) in the desired molar ratio were added and stirred for 2.5 min. Prepared nanoparticles were then concentrated and washed using 50 kDa Amicon Ultra filters and impregnated onto TiO_2_ maintaining a total metal loading of 1.5%.

Plain PEO solutions were prepared by simply dissolving the polymer powder in water under magnetic stirring at room temperature until forming a homogeneous solution.

PEO/PEGDA blends without the catalyst were prepared as follows: (i) dissolution of the PI into the right amount of PEGDA (liquid oligomer) to obtain a PEGDA/PI solution; (ii) preparation of a PEO solution using only two thirds of the necessary water amount; and (iii) addition of the PEGDA/PI solution to the PEO solution to obtain the final PEO/PEGDA/PI solution, using the remaining one third of water for transferring the PEGDA/PI solution completely.

The solution containing the catalyst supported on titania (Au_6_Pd_1_/TiO_2_) was prepared by adding the catalyst (13% wt with respect to the overall polymeric fraction) to the preformed PEO/PEGDA/PI solution. Before their electrospinning, the solutions containing the PI were kept out of the light by covering the vials with aluminum foil to prevent any photo-crosslinking.

The nanofibrous mats were produced using an electrospinning machine (Lab Unit, Spinbow s.r.l., Bologna, Italy) equipped with two 5 mL syringes joined via Teflon tubing to translating needles (length 55 mm, internal diameter 0.84 mm). A drum rotating with a tangential speed of 0.39 m/s, covered with polyethylene-coated paper, was used as a collector. The electrospinning process was conducted in an air-conditioned room, with 23–25 °C and relative humidity (RH) ranging from 23 to 27%. The electrospun mats had final dimensions of 15 × 25 cm and a thickness of 40–45 μm (measured with an analog indicator (Borletti, Italy), under 360 g/m^2^ pressure).

Details of solutions, blends, and electrospinning process parameters are reported in Table 1 and Table 2.

### 2.3. Mats’ Characterization

The mats’ morphological characterization was evaluated using scanning electron microscopy (Phenom ProX, ThermoFisher Scientific, Waltham, MA, USA), recording the images at 10 kV. All analyzed surfaces had previously been gold-coated using a Quorum SC7626 sputter coater (180 s, 18 mA). The average fiber diameters were obtained from at least 100 measurements, manually taken from single nanofibers using the Photoshop measurement tool.

The mats’ thermal properties were assessed via differential scanning calorimetry (DSC, model Q2000 equipped with an RCS cooling system, TA Instruments, New Castle, DE, USA). Samples of 8–10 mg were heated/cooled at 20 °C/min under a nitrogen atmosphere.

The degree of crystallinity (χ_c_) was calculated according to Equation (1):

(1) $$\chi_{c}\;\left(\%\right)=\frac{\Delta H_{m}^{exp}}{\Delta H_{m}^{100\%\;cryst}}\cdot 100$$

where $\Delta H_{m}^{exp}$ is the experimental PEO melting enthalpy, and $\Delta H_{m}^{100\%\;cryst}$ is the melting enthalpy of a theoretical 100% crystalline PEO. The $\Delta H_{m}^{100\%\;cryst}$ of PEO was estimated to be 203–205 J/g [55,56]; here, a value of 204 J/g was considered for the χ_c_ calculation. All of the χ_c_ values were normalized to the actual PEO content.

### 2.4. Water Resistance Tests on Photo-Crosslinked Mats

The photo-crosslinked mats, using UV radiation at 254 nm, underwent water washings (1 h at room temperature) to evaluate their resistance in an aqueous environment. The effect of water on the nanofibers’ morphology was assessed via SEM.

### 2.5. Catalytic Test Using a Water Phase Model Reaction

The catalytic activity of the PEO-based membrane was evaluated by testing it in the reduction of 4-nitrophenol (4-NP) to 4-aminophenol (4-AP), a water phase model reaction that uses NaBH_4_ as a reducing agent. The reaction was followed in situ with a double beam spectrophotometer (Lambda 19 from Perkin Elmer, Sheltor) at the wavelength of 400 nm (maximum absorption of 4-NP in basic ambient). The molar ratios 4-NP:Au_6_Pd_1_ and 4-NP:NaBH_4_ were 4.09 and 6.25 × 10^−4^, respectively. The catalytic test on the membranes was compared with the ones accomplished with Au_6_Pd_1_/TiO_2_ in powder.

The kinetic constant of the two catalysts was calculated as the slope of the linear part (time of reaction when the reaction is actually taking place, excluding the induction time and the post-reaction time) of the curves $\ln\left(\frac{A}{A_{0}}\right)$ vs. time of reaction.

## 3. Results and Discussion

Immobilizing a catalyst on a supporting material may be preferable over directly using a catalyst powder, which is almost necessary when dealing with a flow reactor. Nanofibers may be ideal supporting substrates, provided that reagents are able to diffuse inside the nanofiber and reach the catalyst. Here, PEO was chosen for its low *T_g_* and hydrophilicity, while the crosslinkable PEGDA was selected to enable the overall fibrous structure retention in an aqueous environment.

**Scheme 1:**
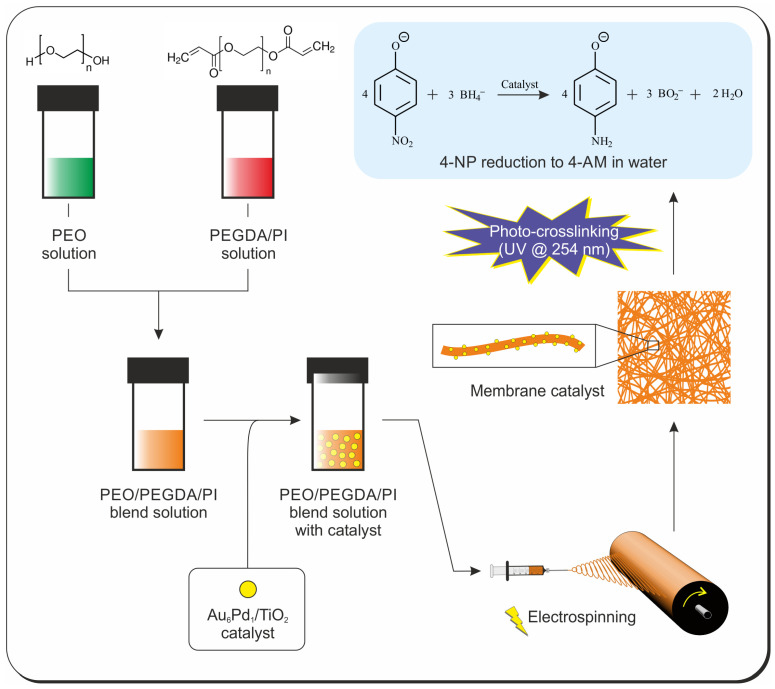
Overview of the work: preparation of PEO solutions and PEO-based blends, processing via electrospinning, mats’ characterization, and mat testing catalyst membranes in the reduction of 4-nitrophenol (4-NP) to 4-aminophenol (4-AP).

Scheme 1 provides a general overview of the work, from the preparation of solutions/blends to the final test of the nanofibrous membrane in the reduction of 4-nitrophenol (4-NP) to 4-aminophenol (4-AP), taken as a “model reaction”.

### 3.1. Preliminary Solutions and Electrospinnability of Different PEO Molecular Weights

The PEO polymer is commercially available in a wide variety of molecular weights (M_w_s). To achieve the main goal, that is, the best fibrous morphology retention in water, the use of high M_w_s should be preferred. Indeed, the presence of higher macromolecular chains should hamper polymer solubility. However, the best balance between polymer viscosity and processability via the electrospinning process should be pursued, taking into account the overall best output (quality of fibers’ morphology, process stability, and productivity).

To produce preliminary PEO solutions (Table 1), using water as a solvent, and to carry out electrospinning tests, three different M_w_s were selected: 100, 400, and 1000 kDa.

**Figure 2:**
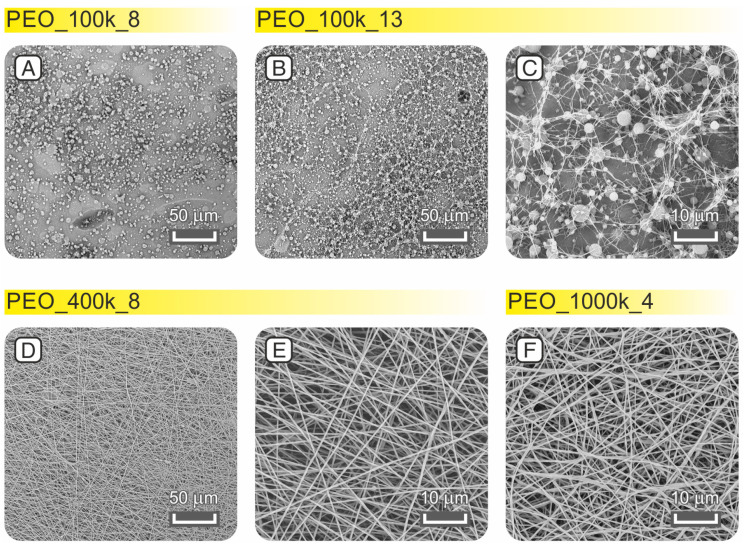
SEM images of PEO solutions processed via electrospinning: effect of molecular weight and solution concentration. (**A**) PEO 100 kDa, 8% wt solution; (**B**,**C**) PEO 100 kDa, 13% wt solution; (**D**,**E**) PEO 400 kDa, 8% wt solution; (**F**) PEO 1000 kDa, 4% wt solution.

The electrospinning of the lowest PEO molecular weight (100 kDa) does not allow for the production of nanofibers. Indeed, electrospray is the predominant phenomenon, leading to the production of a film and pearly particles (Figure 2A, PEO_100k_8). Raising the solution concentration to 13% wt does not yield satisfactory results, still obtaining a combination of fibers, beads, and pearly drops (Figure 2B,C, PEO_100k_13).

Using 400 kDa PEO gives a completely different output: thin (383 ± 56 nm), continuous, and bead-free nanofibers are positively produced (PEO_400k_8, Figure 2D,E). Even the 1000 kDa polymer generates nanofibers (407 ± 42 nm) with a good morphology (PEO_1000k_4, Figure 2F). The high molecular weight should favor the morphology retention in an aqueous environment; however, the electrospinning of such a solution requires a reduced concentration (4% wt) to avoid an unviable solution viscosity, besides the application of a very low flow rate (0.2 mL/h) which results in poor productivity.

Therefore, based on this evidence, the 400 kDa PEO appears to be the most suitable material for nanofibrous mat production, provided that such a M_w_ will allow the formation of PEO/PEGDA blends and nanostructure retention in a water environment.

### 3.2. PEGDA Crosslinking Assessment

A sufficiently high M_w_ PEO may help to maintain the fibrous structure but cannot avoid its dissolution in water. Therefore, the formation of covalent bonds via polymer crosslinking is fundamental. For this reason, the addition of a crosslinkable PEO-like material, that is, poly(ethylene glycol) diacrylate (PEGDA), was chosen for its intrinsic blending ability with PEO, owing to chemical backbone identity. Indeed, exploiting the double bonds of PEGDA, it may be possible to form an insoluble net able to retain the uncrosslinked PEO macromolecules.

**Figure 3:**
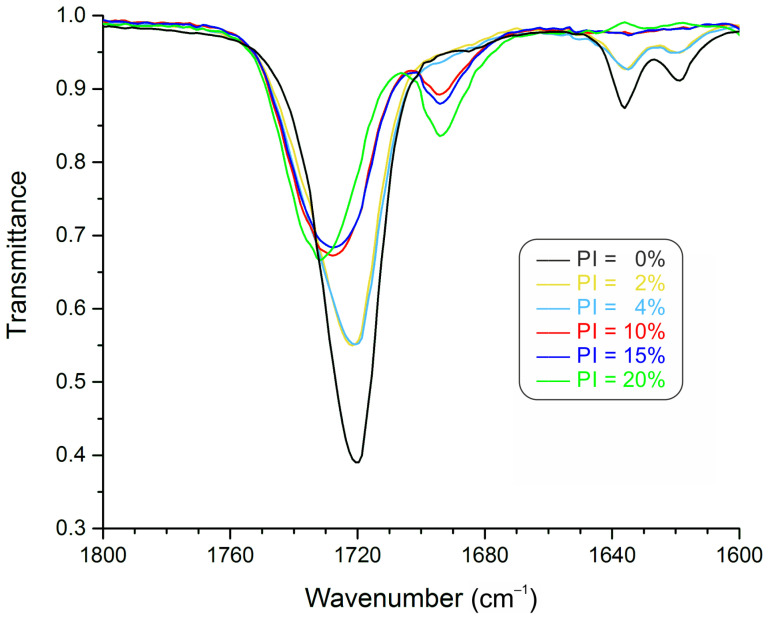
ATR-IR spectra of PEGDA/PI solutions containing different PI amounts, photo-crosslinked for 30 min.

Before proceeding with PEO/PEGDA formulations, the effective PEGDA crosslinking via UV irradiation was assessed. In particular, several PEGDA/photoinitiator combinations were tested, containing from 2 to 20% mol of PI. The photo-crosslinking was evaluated by carrying out IR measurements (Figure 3).

Up to 4% mol of PI, the recorded spectra are similar to the one of PEGDA, indicating a minimal reaction occurrence. This fact may happen for two reasons, which may also be concomitant: (i) the PEGDA oligomer contains a crosslinking inhibitor to favor the shelf-life, and (ii) the environmental oxygen. In the first case, the inhibitor acts by deactivating the radicals promoted by the UV radiation, while in the second one, the oxygen itself acts as an “inhibitor”. It is worth mentioning that the photo-crosslinking was carried out in environmental conditions to mimic the real ambient conditions that will face PEO/PEGDA membranes.

The recorded IR spectra sensibly change starting from 10% mol PI concentration. The 1694 cm^−1^ peak appears, probably due to the stretching of the PEGDA carbonyl conjugated with the benzenic ring of the PI molecule. The C=O peak at 1720 cm^−1^ moves toward higher wavenumbers due to the lack of conjugation with the vinyl double bond of the acrylic residue upon its polymerization. The highest PI concentration (20% mol) seems to promote the reaction the most (highest peak shift to 1732 cm^−1^), while 10 and 15% mol of PI cause a shift to 1728 cm^−1^; the shift of solutions with 2 and 4% mol of PI is between 1720 and 1728 cm^−1^. However, a very high PI concentration may negatively affect the polymer’s mechanical properties. For this reason, 10% mol PI concentration was considered the best option, and it was adopted for producing PEO/PEGDA/PI blends.

**Figure 4:**
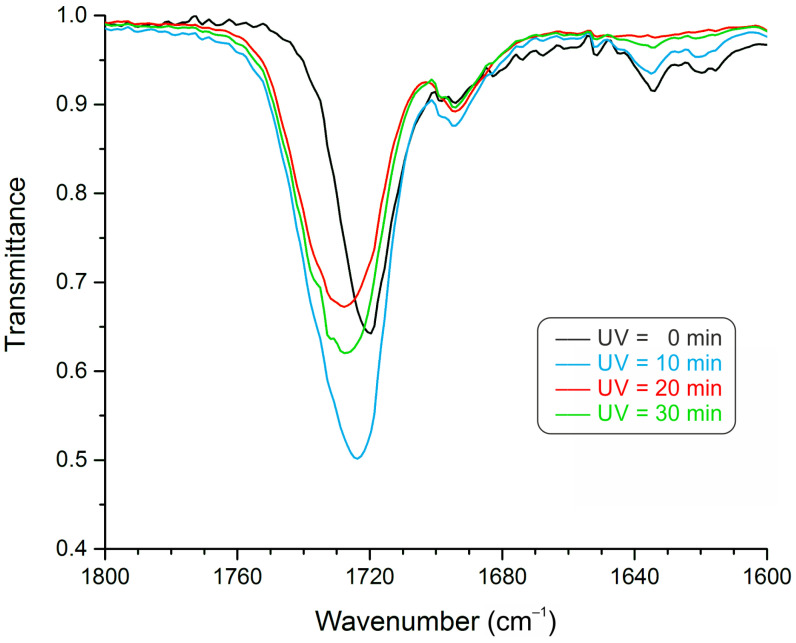
Effect of UV irradiation time on PEGDA crosslinking reaction with 10% mol of PI.

Before proceeding, the effect of UV irradiation time was also evaluated (Figure 4).

Moving from a non-irradiated material (0 min) to 20 min of irradiation time, there is a gradual shift of the C=O peak (1720 cm^−1^ for 0 min, 1724 cm^−1^ for 10 min, and 1728 cm^−1^ for 20 min), besides a concomitant reduction in the C=C signal intensity. The irradiation for 30 min does not further boost the reaction.

### 3.3. PEO/PEGDA Blends: Solutions, Electrospinning, and Morphology Retention Test in Water

Solution blends of PEO and PEGDA were first produced by mixing them 1:1 by weight, using PEO 400 kDa according to the outcomes discussed in Section 3.1 (Table 2).

**Figure 5:**
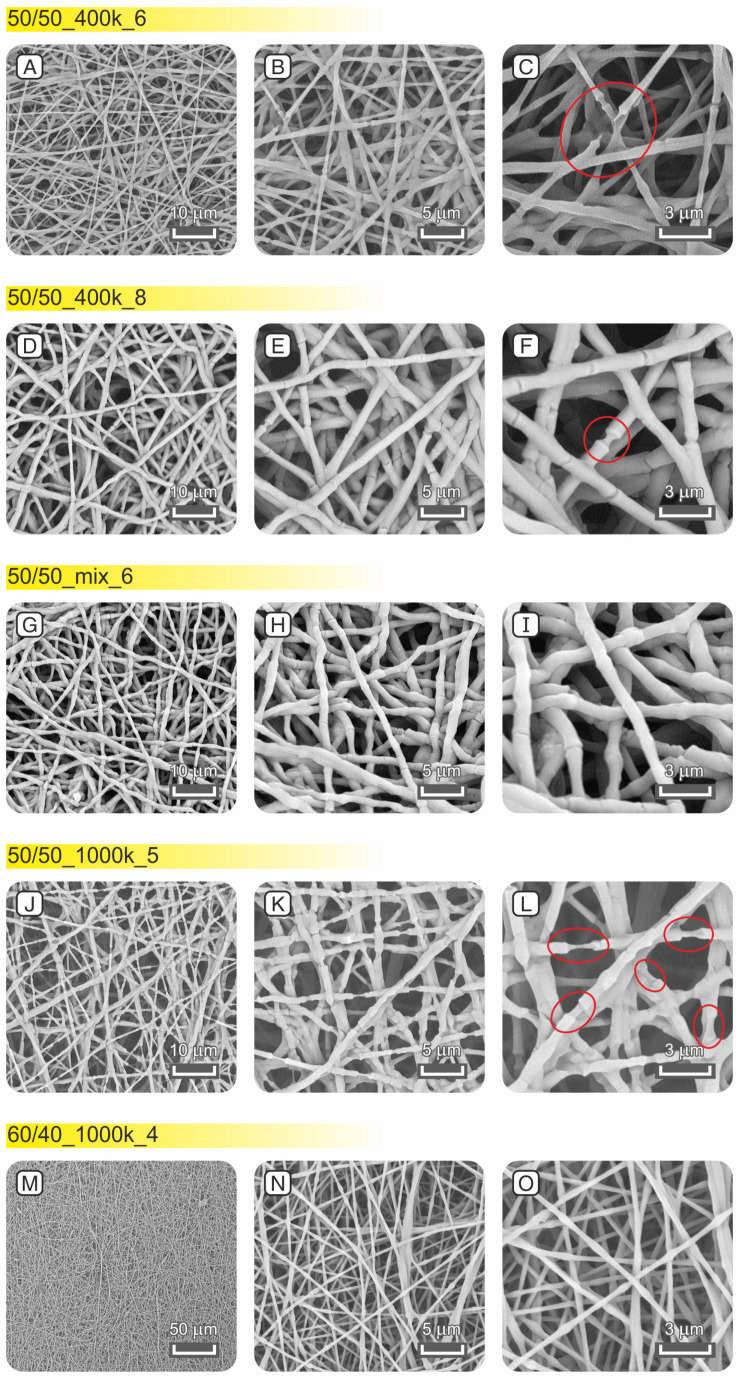
SEM images of PEO/PEGDA blend solutions processed via electrospinning; effect of PEO molecular weight, PEO/PEGDA ratio, and solution concentration. (**A**–**C**) blend 50/50 wt, PEO 400 kDa, 6% wt solution; (**D**–**F**) blend 50/50 wt, PEO 400 kDa, 8% wt solution; (**G**–**I**) blend 50/50 wt, PEO 400 and 1000 kDa, 6% wt solution; (**J**–**L**) blend 50/50 wt, PEO 1000 kDa, 5% wt solution; (**M**–**O**) blend 60/40 wt, PEO 1000 kDa, 4% wt solution. Red circles indicate fiber interruption or necking phenomenon.

The fibers’ morphology is not optimal (Figure 5A–F); indeed, there are evident and frequent necking phenomena along the nanofibers, which may also lead to fiber interruption (Figure 5C). This may be due to the presence of a too-high PEGDA fraction (50% wt) that prevents the polymeric blend from having sufficient viscosity. However, the problem may also arise from a low solution viscosity resulting in an unstable polymeric jet development during the electrospinning process. In the latter case, the electrospinning of a more concentrated solution should be beneficial. In the present case, raising the concentration from 6% to 8% wt indeed prevents the fiber interruptions, while the necking phenomenon is still present (Figure 5F). Even if there is a slight enhancement of the fiber morphology, this appears insufficient for the work’s aim. Moreover, raising the concentration leads to higher diameters, resulting in near microfibers (833 ± 133 nm for 50/50_400k_8 vs. 438 ± 112 nm for 50/50_400k_6).

Since higher blend concentrations (10 and 12% wt) cannot be processed via electrospinning, we tested a blend whose PEO fraction was composed of 70% wt of PEO 400 kDa and 30% wt of PEO 1000 kDa (50/50_mix_6). The highest M_w_ polymer, indeed, may help to obtain a better fiber morphology by increasing the electrospun jet viscosity (and in turn the entanglements’ form ability). The morphology improved (50/50_mix_6, Figure 5G–I) with respect to mere 400 kDa PEO mats (Figure 5A–F); however, the fiber quality is still insufficient, besides large diameters laying almost in the micrometer range (903 ± 186 nm).

A further attempt was made by electrospinning only 1000 kDa PEO in blend with PEGDA, keeping a 50:50 PEO:PEGDA ratio (50/50_1000k_5). Due to the high molecular weight, the blend concentration was slightly reduced to 5% wt (higher concentrations lead to gel formation). The electrospun material, displayed in Figure 5J–L, is characterized by nanofibers (664 ± 124 nm) but affected, again, by abundant and “long” neckings having almost a halved diameter (295 ± 83 nm).

**Figure 6:**
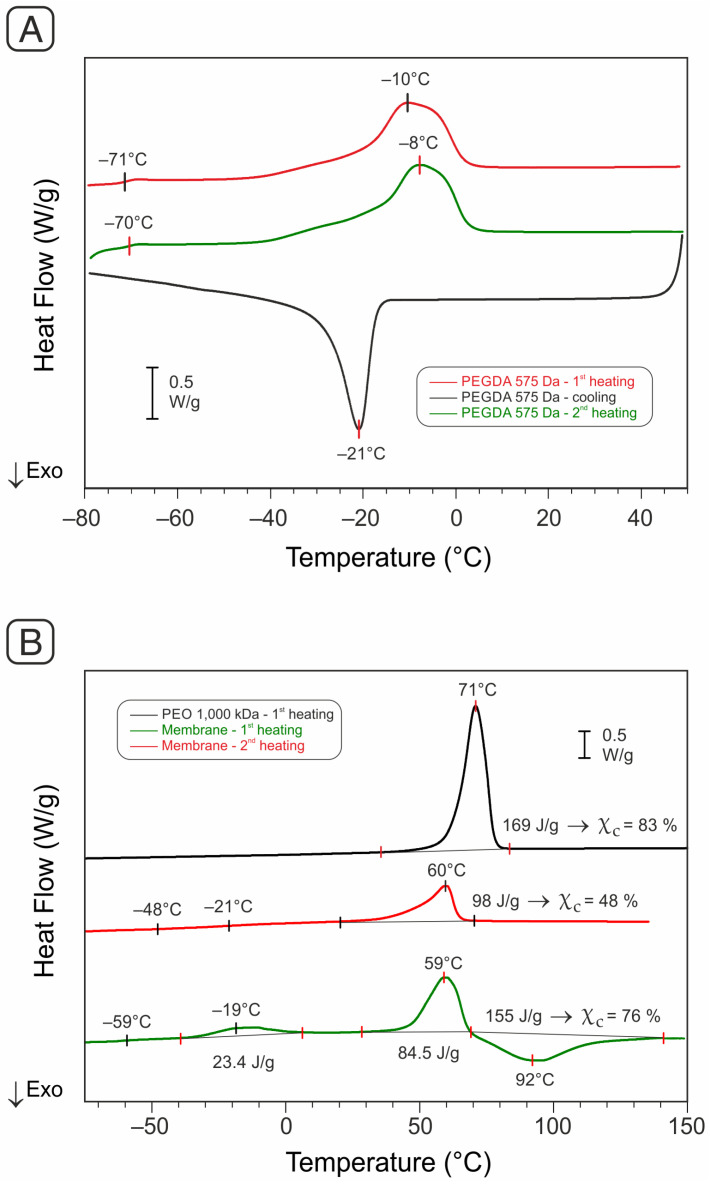
(**A**) DSC analysis of PEGDA; (**B**) DSC analysis of PEO 1000 kDa, and photo-crosslinked 60/40_1000k_4_PI-10 mat after 30 min of UV irradiation time (the use of 5% mol of PI gives similar thermograms). For the membrane, the melting enthalpy is normalized on the PEO fraction.

A significant morphology enhancement is achieved by increasing the PEO fraction from 50 to 60% wt in the PEO/PEGDA blend and slightly lowering the total polymer concentration from 5% to 4% wt (60/40_1000k_4, Figure 5M–O). Here, the nanofibers (353 ± 77 nm) are continuous, with no necking (only some swelling of the nanofibers occurs). For this reason, the 60/40_1000k_4 blend was chosen as the best candidate for producing the catalytic nanofibrous mat. Before adding the catalytic active phase, a preliminary evaluation of the morphology retention of crosslinked mats in water was carried out by testing electrospun membranes containing a 5 and 10% mol photoinitiator; their thermal behavior was assessed via DSC analysis (Figure 6).

The photocrosslinked PEO/PEGDA mat (60/40_1000k_4_PI-10) displays four key signals:

(1) A stepwise signal at −60 °C accounting for PEGDA *T*_g_;
(2) A low-T endothermic peak, centered at −16 °C, due to the PEGDA melting;
(3) A high-T endothermic peak, centered near 60 °C, due to PEO melting;
(4) An exothermic peak starting just after PEO melting, accounting for residual PEGDA crosslinking.

The χ_c_ of PEO in the PEO/PEGDA blend is still high (76%); the great amount of the crystal phase is undoubtedly useful to confer good mechanical properties to the electrospun membrane, helping it to maintain the nanofibrous structure besides the crosslinked material.

After the first heating, the glass transition moves to −21 °C, while both PEGDA’s melting and crosslinking disappear. These facts indicate that, upon UV curing, the PEGDA crosslinking is not complete.

**Figure 7:**
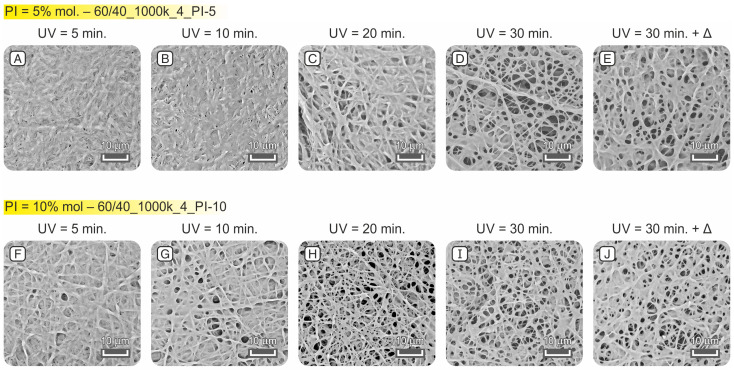
Effect of photoinitiator concentration, UV irradiation time, and eventual thermal treatment on PEO/PEGDA 60:40 mats’ morphology after water washing evaluated via SEM. The mats contain 5% mol (**A**–**E**) and 10% mol (**F**–**J**) of photoinitiator.

The resulting mats’ morphology after washings is presented in Figure 7. The UV photo-crosslinking was carried out for different irradiation times (5, 10, 20, and 30 min) to find the best morphology retention.

At first glance, it appears that mats containing 10% mol photoinitiator (Figure 7F–J) behave better than the counterpart with a halved amount of it (Figure 7A–E). In the latter case, for 10 min or shorter irradiation times, the mat morphology is wholly lost (Figure 7A,B), while the best fibrous structure occurs when photo-crosslinking is carried out for 30 min (Figure 7D). The mats with 10% mol of photoinitiator generally behave better than the previous ones, even when low UV irradiation times are applied. Indeed, just 5 min is sufficient to retain a coarse fibrous structure (Figure 7F). The behavior improves by doubling the irradiation time (Figure 7G), and the best condition is achieved by irradiating for 20 min (Figure 7H). Higher irradiation times lead to a worsening structure (30 min, Figure 7I).

Considering the DSC analysis, thermal activation can further boost the PEGDA crosslinking. Indeed, photoinitiators can also act as thermal initiators, given that sufficient thermal energy is provided. For these reasons, an attempt to further improve the mats’ crosslinking was carried out by applying a thermal treatment (20 min at 120 °C) after UV curing. The preliminary UV curing is required to avoid PEO melting and a loss of the nanofibrous morphology when the temperature is raised. In both mat types (60/40_1000k_4_PI-5, Figure 7E, and 60/40_1000k_4_PI-10, Figure 7J), the thermal crosslinking does not further improve the nanofibrous structure retention in water (actually, it has a negative effect with respect to UV irradiation time of 30 min). However, UV-promoted crosslinking is able to avoid the complete destruction of the nanofibrous structure, which would occur with uncrosslinked materials.

It can be concluded that 5% mol of photoinitiator is insufficient to guarantee acceptable structure retention, and 10% mol of PI should be used. Regarding the UV irradiation time, 20 min of photo-crosslinking results in the best compromise between sufficient morphology retention and possible material performance lowering due to eventual UV degradation.

**Figure 8:**
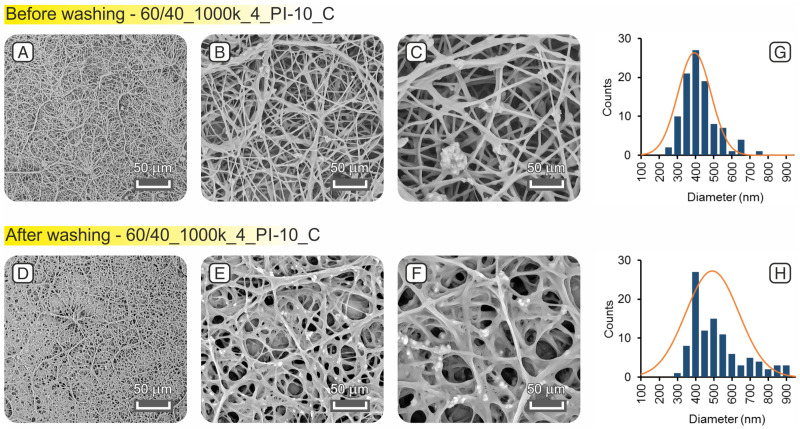
Catalyst-containing PEO/PEGDA 60:40 photo-crosslinked nanofibers’ morphology; mats before (**A**–**C**) and after (**D**–**F**) water resistance test. (**G**,**H**) display the fibers’ diameter distributions before and after the water treatment.

Adding the catalyst supported on titania (Au_6_Pd_1_/TiO_2_) to the solution blend (60/40_1000k_4_PI-10_C) does not negatively affect the electrospinning process or the nanofibrous structure output. Figure 8A–C shows the mat’s morphology obtained after the photo-crosslinking step, carried out for 20 min. The nanofibers have 393 ± 91 nm diameters, resulting in them being statistically comparable to the ones of the same nanofibers without a catalyst (353 ± 77 nm, 60/40_1000k_4).

The resulting mat’s morphology after the water resistance test is displayed in Figure 8D–F. The main structure is maintained, and the fibers still have diameters ranging in the nanometric dimension (491 ± 142 nm).

### 3.4. Evaluation of the Catalytic Effect of PEO/PEGDA Nanofibers

The membrane displaying the best stability (60/40_1000k_4_PI-10) was then modified by adding into the solution blend the active phase, that is, gold-palladium (Au_6_Pd_1_) nanoparticles supported on titania (TiO_2_).

**Scheme 2:**
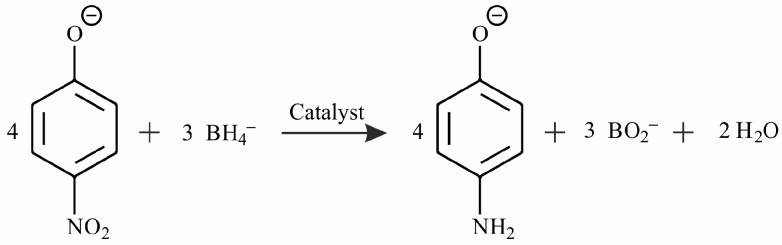
Water phase model reaction (reduction of 4-NP to 4-AP) used to test the PEO/PEGDA catalytic membrane.

The catalytic activity of the catalytic membrane (60/40_1000k_4_PI-10_C) was evaluated, testing it in the reduction of 4-NP to 4-AP, a water phase model reaction that uses NaBH_4_ as a reducing agent (Scheme 2).

This reaction is considered a model reaction for easy screening of the catalytic activity of nanomaterials for several reasons, e.g., it can be followed in situ with a spectrophotometer, there are no secondary reactions, and the reaction is kinetically slow; therefore, without a catalyst, the reagents are stable [57]. Moreover, if the reaction is conducted in an excess condition of NaBH_4_ so that it is possible to assume its concentration as constant, the kinetic can be evaluated with a first-order kinetic equation.

Initially, the stability of 4-NP in the reaction conditions without the presence of the catalyst was verified. The absorbance of the solution at 400 nm remains constant even 60 min after the preparation of the solution, implying that no reaction is taking place (Figure S1, Supporting Information).

The catalytic test on the membrane was compared with the ones accomplished with Au_6_Pd_1_/TiO_2_ in powder. The comparison is carried out in terms of the length of induction and kinetics constant. The former is the time required for the reaction to begin, which can be correlated with the time required for the reagents to diffuse from the bulk of the solution to the catalytic sites. In this particular reaction, the induction time is a typical feature even when no diffusion obstacle is present. Indeed, it can be due to both the formation of a thin oxidated layer on the nanoparticle catalyst surface that must be removed to activate the catalyst, and the presence of dissolved oxygen that can react with NaBH_4_ [57].

**Figure 9:**
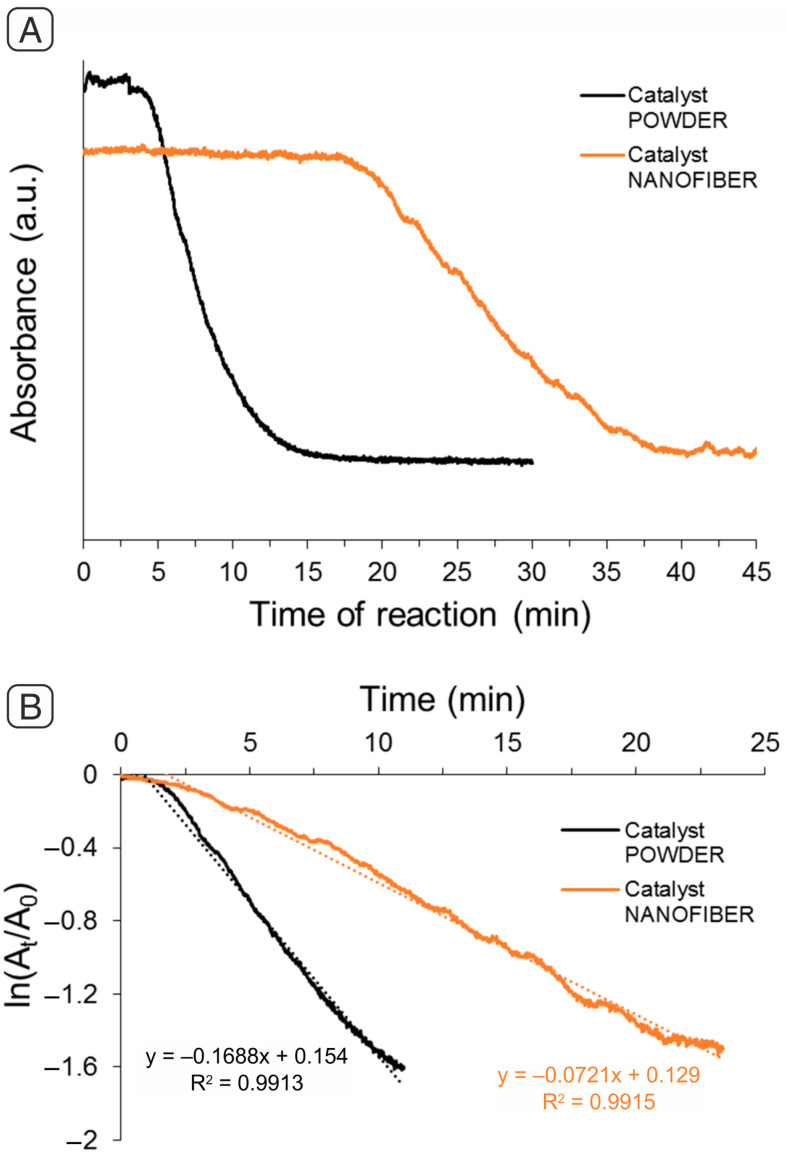
(**A**) UV-vis spectra recorded at 400 nm: comparison between model reaction carried out using catalyst powder and catalyst supported on nanofibers. (**B**) Kinetic elaboration of UV-vis data.

When the catalyst is directly inserted into the membrane, an increase in the induction time from 3 to 17 min occurs (Figure 9A). This is clearly related to the greater diffusional barrier represented by the presence of the polymeric PEO/PEGDA matrix. Nevertheless, it must be considered that the reaction is accomplished without stirring the solution, and the mixing is just due to the formation of H_2_. Considering that the membrane is designed to be applied in a continuous flow-through setup, this results in the unstirred condition being promising and confirms the remarkable contactor capability of the membrane in the aqueous phase.

The membrane is less active than the powder, though the kinetic constant of the two catalyst types is comparable: 0.07 min^−1^ and 0.17 min^−1^, respectively (Figure 9B). Incorporating the catalyst into the electrospun membrane probably led to a partial deactivation of part of the active sites, partially slowing the reaction but maintaining the capability of promoting the reaction. Hence, the results highlight that the design concept of the membrane is promising for water phase application at low temperature.

## 4. Conclusions

In the present work, PEO/PEGDA blends containing Au_6_/Pd_1_ supported on TiO_2_ were electrospun to produce polymeric catalytic membranes for water phase and low-temperature reactions. The membranes underwent photo-crosslinking by UV radiation to attain a stable nanofibrous morphology in a water environment.

In particular, the PEO molecular weight (M_w_), PEO:PEGDA ratio, photoinitiator (PI) concentration, and UV irradiation time were optimized to obtain water-resistant PEO-based membranes.

Using a high M_w_ PEO (1000 kDa), a 60:40 wt PEO:PEGDA ratio in the presence of a 10% mol PI, and a UV irradiation time of 20 min leads to the best result in terms of the nanostructure’s morphology retention in water.

The reduction of 4-nitrophenol (4-NP) to 4-aminophenol (4-AP), taken as a model reaction, was used to test the catalytic ability of PEO-based nanofibrous mats, demonstrating the potential use of such crosslinked polymeric membranes in water phase reactions.

## Figures and Tables

**Table 1:** Characteristics of aqueous PEOs’ solutions and electrospinning parameters.

Solution	Concentration (%wt)	Flow Rate (mL/h)	Potential (kV)	Distance (cm)
PEO_100k_8	8	0.5	18	15
PEO_100k_13	13	0.5	21	15
PEO_400k_8	8	1.2	15	15
PEO_1000k_4	4	0.2	18	15

**Table 2:** PEO/PEGDA solution blends and electrospinning parameters.

Solution Blend	PEO M_w_ (Da)	PEO/PEGDA wt Fraction	Polymer Conc. (% wt)	PI (% mol)	Flow Rate (mL/h)	Electric Potential (kV)	Distance (cm)
50/50_400k_6	400k	50:50	6	0	0.8	15	15
50/50_400k_8	400k	50:50	8	0	0.8	15	15
50/50_mix_6	70% wt 400k 30% wt 1000k	50:50	6	0	0.8	18	20
50/50_1000k_5	1000k	50:50	5	0	0.8	14	15
60/40_1000k_4	1000k	60:40	4	0	0.6	16	14
60/40_1000k_4_PI-5	1000k	60:40	4	5	0.6	16	14
60/40_1000k_4_PI-10	1000k	60:40	4	10	0.6	16	14
60/40_1000k_4_PI-10_C	1000k	60:40	4	10	0.5	15	12

## Data Availability

The data presented in this study are available upon request from the corresponding author.

## Supplementary Materials

The following supporting information can be downloaded at https://www.mdpi.com/article/10.3390/membranes13020212/s1: Figure S1: UV-vis spectra recorded at different times during the water phase model reaction: (A) using the PEO/PEGDA membrane 60/40_1000k_4_PI-10 (without catalyst), and (B) using the mat containing the catalyst (60/40_1000k_4_PI-10_C).
