# Supplementary material for: Water-Resistant Photo-Crosslinked PEO/PEGDA Electrospun Nanofibers for Application in Catalysis
Source: Membranes (Basel). 2023 Feb 8;13(2):212. doi: 10.3390/membranes13020212 (PMC9968077; doi:10.3390/membranes13020212)
Supplement: Supplementary file 1 [file membranes-13-00212-s001.zip › membranes-2151188-supplementary.pdf]

Supporting Information

Water-resistant photo-crosslinked PEO/PEGDA electrospun nanofibers for application in catalysis

Emanuele Maccaferri [1,2,3,*], Andrea Canciani[1], Laura Mazzocchetti [1,2,3], Tiziana Benelli [1,2,3], Loris Giorgini [1,2,3], and Stefania Albonetti[1,2,3]

[1] Department of Industrial Chemistry "Toso Montanari", University of Bologna, Viale Risorgimento 4, 40136 Bologna, Italy

[2] National Interuniversity Consortium of Materials Science and Technology (INSTM), 50121 Florence, Italy

[3] Interdepartmental Center for Industrial Research on Advanced Applications in Mechanical Engineering and Materials Technology, CIRI-MAM, University of Bologna, Viale Risorgimento 2, 40136 Bologna, Italy

* Correspondence: emanuele.maccaferri3@unibo.it

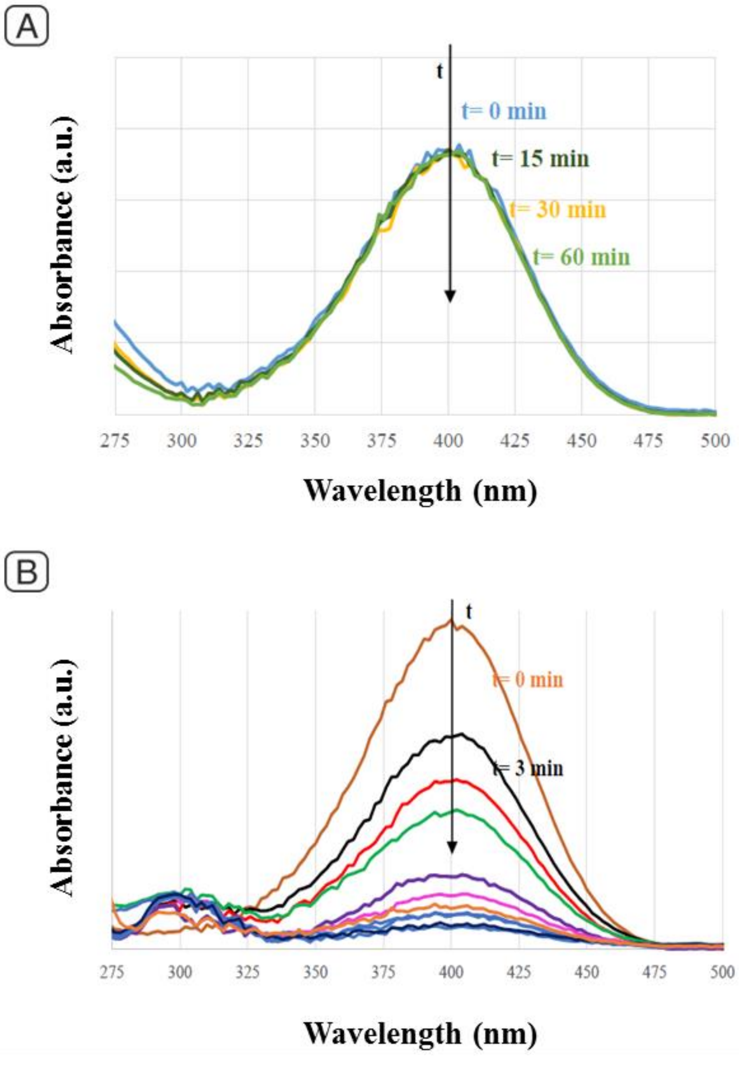

Figure S1. UV-vis spectra recorded at different times during the water-phase model reaction: (A) using the PEO/PEGDA membrane 60/40_1000k_4_PI-10 (without catalyst), and (B) using the mat containing the catalyst (60/40_1000k_4_PI-10_C).
